# LncRNA LOXL1‐AS1 regulates the tumorigenesis and development of lung adenocarcinoma through sponging miR‐423‐5p and targeting MYBL2

**DOI:** 10.1002/cam4.2641

**Published:** 2019-11-23

**Authors:** Wei Li, Biao Zhang, Youchao Jia, Hongyun Shi, Haibo Wang, Qiang Guo, Hefei Li

**Affiliations:** ^1^ Department of Thoracic Surgery Affiliated Hospital of Hebei University Baoding China; ^2^ Department of Medical Oncology Affiliated Hospital of Hebei University Baoding China; ^3^ Department of Radiation Oncology Affiliated Hospital of Hebei University Baoding China

**Keywords:** LOXL1‐AS1, lung adenocarcinoma, miR‐423‐5p, MYBL2

## Abstract

Lung adenocarcinoma (LUAD) is the most common form of malignant tumor and closely correlated with high risk of death worldwide. Accumulating researches have manifested that long noncoding RNAs (lncRNAs) are deeply involved in the progression of multiple cancers. LncRNA LOXL1 antisense RNA 1 (LOXL1‐AS1) was identified as an oncogene in several cancers, nonetheless, its biological effect and regulatory mechanism have not been explained in LUAD. Our present study suggested that LOXL1‐AS1 expression was considerably increased in LUAD tissues and cells. Moreover, LOXL1‐AS1 deficiency notably hampered cell proliferation and migration as well as dramatically facilitated cell apoptosis. Through molecular mechanism assays, LOXL1‐AS1 was identified as a cytoplasmic RNA and acted as a sponge of miR‐423‐5p. Furthermore, MYBL2 was targeted and negatively modified by miR‐423‐5p. Rescue experiments revealed that MYBL2 knockdown could counteract miR‐423‐5p repression‐mediated enhancement on the progression of LOXL1‐AS1 downregulated LUAD cells. More importantly, MYBL2 was discovered to interact with LOXL1‐AS1 promoter, indicating a positive feedback loop of LOXL1‐AS1/miR‐423‐5p/MYBL2 in LUAD. These findings manifested the carcinogenic role of LOXL1‐AS1 and LOXL1‐AS1/miR‐423‐5p/MYBL2 feedback loop in LUAD, which could be helpful to explore effective therapeutic strategy for LUAD patients.

## Graphical abstract

(A) LOXL1 antisense RNA 1 (LOXL1‐AS1) is upregulated in lung adenocarcinoma tissues and cells. (B) LOXL1‐AS1 facilitates cell proliferation and migration as well as suppresses cell apoptosis. (C) LOXL1‐AS1 acts as a sponge for miR‐423‐5p.

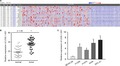

## 1 INTRODUCTION

Lung adenocarcinoma (LUAD), a main type of non‐small cell lung cancer, has become a popularly aggressive cancer and ranks the first in terms of tumor‐related deaths through all over the world.^1^, ^2^, ^3^ Patients suffered from LUAD at advanced stage are usually diagnosed with the metastasis.^4^ Due to a lack of effective treatment, the prognostic of LUAD patients is tremendously poor.^5^ Therefore, there is an exigent necessity to probe novel regulatory mechanism of LUAD and to investigate underlying therapeutic targets for LUAD patients.

Long noncoding RNAs (lncRNAs) are a type of transcripts that lengths are over 200 nucleotides and limited to encode proteins and related to biological processes.^6^, ^7^, ^8^ A growing number of lncRNAs exerted their regulatory roles in various tumors. LncRNA CHRF, a sponge of miRNA‐10b, promoted cell proliferation and epithelial‐mesenchymal transition (EMT) process in prostate cancer.^9^ LncRNA ZEB1‐AS1 mediated the miR‐200c/141‐ZEB1 axis to facilitate tumor growth and cell metastasis in glioma cancer.^10^ LncRNA SNHG14, which was identified as an oncogene, boosted the development of cervical cancer via regulating miR‐206 and targeting YWHAZ.^11^ Previous investigations also indicated that a variety of lncRNAs were participated in the regulation of LUAD development. LINC00324 targeted miR‐615‐5p/AKT1 axis to promote the tumor process of LUAD.^12^ LncRNA LINC00460 promoted tumor growth and resulted to a terrible prognosis in LUAD by completely sponging miR‐302c‐5p and upregulating FOXA1 expression.^13^ Overexpressed SNHG6 in LUAD promoted cell proliferation and metastasis through regulating E2F7 expression and sponging miR‐26a‐5p.^14^ LOXL1 antisense RNA 1 (LOXL1‐AS1) has been confirmed as an oncogene and its regulatory mechanism in other cancers has been explored. In medulloblastoma, LOXL1‐AS1 enhances its proliferation and metastasis via the activation of PI3K pathway.^15^ Nevertheless, its biological role and molecule mechanism in LUAD remain largely unknown.

Here, we explored the functional role and potential mechanism of LOXL1‐AS1 in LUAD, and the interaction between LOXL1‐AS1 and MYBL2 feedback loop was also studied. It was found that LOXL1‐AS1 accelerates tumor progression of LUAD through sponging miR‐423‐5p and upregulating MYBL2, which meant that LOXL1‐AS1 could be a regulator in LUAD development.

## 2 MATERIALS AND METHODS

### 2.1 Cell tissues

Forty patients who underwent surgery at Affiliated Hospital of Hebei University from February 2016 to March 2017 provided the LUAD tissues and matched adjacent normal tissues. They all did not receive any preoperative treatment. For extracting RNA, the specimens were collected and promptly stored at −80°C after surgery. This research was conducted with the approval from ethics committee of Affiliated Hospital of Hebei University. Before surgery, all patients signed the written informed consents.

### 2.2 Cell culture

Human bronchial epithelial cell (BEAS‐2B) and human LUAD cells (H1299, H1975, A549, SPC‐A1) were obtained from ATCC. All cells were grown in Dulbecco's modified Eagle's medium (DMEM; Invitrogen) containing 10% fetal bovine serum (FBS; Invitrogen), 1% penicillin/streptomycin (Sigma‐Aldrich) in the humidified atmosphere at 37°C with 5% CO_2_.

### 2.3 Cell transfection

Specific shRNAs against LOXL1‐AS1 (sh‐LOXL1‐AS1#1 and sh‐LOXL1‐AS1#2) or MYBL2 (sh‐MYBL2#1 and sh‐MYBL2#2) and their corresponding sh‐NC were attained from Genechem. Besides, miR‐423‐5p mimics, miR‐423‐5p inhibitor, NC mimics, and NC inhibitor were designed by GenePharma. Above plasmids were transfected into A549 or SPC‐A1 cells, respectively. Transfection was carried out for 48 hours via Lipofectamine 2000 (Invitrogen).

### 2.4 Quantitative real‐time PCR (qRT‐PCR)

RNAiso Plus (Takara) was adopted for the extraction of total RNA. Synthesis of cDNA was carried out afterwards. SYBR Premix Ex Taq II Kit (TaKaRa) with a Light Cycler System was utilized for conducting Quantitative PCR. GAPDH or U6 was applied to normalize the RNA input via $2^{-\Delta \Delta C_{t}}$ method.

### 2.5 Cell viability assay

Cell counting kit‐8 (CCK‐8; Dojindo) assay was carried out to explore cell viability. At first, transfected A549 or SPC‐A1 cells were planted into 96‐well plates (1 × 10^4^ cells/well), and each well was supplemented with CCK‐8 reagent. After incubating for additional 2 hours at 37℃, the absorbance (OD) was determined by the Thermo‐max microplate reader (Bio‐Tek).

### 2.6 Colony formation assay

A549 or SPC‐A1 cells after transfection were collected, and then seeded to 6‐well plates at a density of 1 × 10^5^ cells per well. After culturing for 5 days, cells were stained with 1% crystal violet (Sigma‐Aldrich) for 20 minutes. After washing with phosphate buffer saline (PBS; Thermo Fisher Scientific) thrice, the efficiency of clone formation was calculated by DMi8 S Platform Live cell microscope (Leica Microsystems).

### 2.7 TUNEL staining assay

Cell apoptosis was assessed by TUNEL staining assay using the In Situ Cell Death Detection Kit (Roche) following the recommended guidelines. Biologic coloring agent of DAPI (Haoran Biotechnology) or Merge (Gene‐denovo) was applied to dye A549 or SPC‐A1 cells. Relative fluorescence intensity was detected via an EVOS FL microscope (Thermo Fisher Scientific).

### 2.8 Transwell migration assay

Transwell chambers were purchased from Corning Costar. Transfected A549 or SPC‐A1 cells, incubated in complete serum‐free medium, were planted into upper chambers, whereas the lower chamber were filled with complete medium with 10% FBS. Using a cotton swab, the nonmigrated cells were removed. Then, the migrated cells were separately fixed and stained with 4% paraformaldehyde (PFA; The BSZH Scientific) and 0.1% crystal violet. The Olympus 1X71 camera system (Leica) was used to photograph migrated cells.

### 2.9 Separation of cytoplasm and nuclear RNA

A PARIS kit (Thermo Fisher Scientific) was adopted for isolating nuclear and cytoplasmic fractions of A549 or SPC‐A1 cells as per its protocol. Reverse transcription and qRT‐PCR were carried out with extracted RNA.

### 2.10 RNA pull down assay

LOXL1‐AS1 biotin probe and LOXL1‐AS1 no‐biotin probe were constructed by GenePharma. For developing probe‐coated beads, they were cultured with M‐280 Streptavidin magnetic beads (Invitrogen). Then, cell lysates of A549 and SPC‐AS1 cells were incubated with probe‐coated beads at 4°C. Subsequently, RNA complexes combined with the beads were eluted. Levels of microRNAs (miRNAs) were detected with qRT‐PCR.

### 2.11 Luciferase reporter assay

LOXL1‐AS1 wild type (WT), LOXL1‐AS1 mutants (LOXL1‐AS1 MUT‐1, LOXL1‐AS1 MUT‐2 and LOXL1‐AS1 MUT1/2), MYBL2 WT and MYBL2 mutation (MUT) were subcloned into the pmirGLO dual‐luciferase vector (Promega). A549 or SPC‐A1 cells were co‐transfected with LOXL1‐AS1 WT/MUT vectors or MYBL2 WT/MUT and miR‐423‐5p mimics or miR‐423‐5p inhibitor, as well as respective controls (NC mimics or NC inhibitor). Besides, WT or mutation of LOXL1‐AS1 promoter was subcloned into the pGL3 luciferase reporter vector (Invitrogen) to establish promoter WT vector or promoter MUT vector. The two vectors were respectively co‐transfected into A549 or SPC‐A1 cells with sh‐MYBL2#1, sh‐MYBL2#2, or sh‐NC.

### 2.12 RIP assay

Based on the specification offered by the supplier, the EZ‐Magna RIP kit (Millipore, Billerica, MA, USA) was employed to conduct RIP assay. A549 or SPC‐A1 cells were collected via centrifugation and subsequently lysed in RIP lysis buffer (Thermo Fisher Scientific). Cell lysates were subjected to immunoprecipitation with antibody against Ago2 (Millipore) or IgG (Millipore). The immunoprecipitated RNA was harvested and then augmented with qRT‐PCR using the Primer Script RT Master Mix kit (TaKaRa).

### 2.13 Western blot

A549 or SPC‐A1 cells after transfection were lysed in order to extract protein. The concentrations of protein samples were evaluated with the bicinchoninic acid assay kit (Pierce). All extracted proteins were isolated by 10% SDS‐PAGE (Bio‐Rad Laboratories) and later transferred to polyvinylidene fluoride membranes (Millipore). After being treated with primary antibody against MYBL2 (0.8 μg/mL, AV100748, Sigma‐Aldrich) or GAPDH (1/20 000, ab128915, Abcam) and secondary antibodies, immunoreactive bands were exposed via ECL method.

### 2.14 Chromatin immunoprecipitation

The Magna ChIP Kit (Millipore) was adopted for chromatin immunoprecipitation (ChIP) assay. Briefly speaking, after cross‐linked chromatin was initially sonicated to 200‐500‐bp fragments, lysates were immunoprecipitated with antibody against MYBL2 or IgG. Precipitated chromatin DNA was analyzed via qRT‐PCR.

### 2.15 Statistical analysis

GraphPad Prism software (GraphPad) was used to conduct statistical analysis. Data were assessed under Student's *t* test (two groups) or one‐way ANOVA (multiple groups), and shown as mean ± standard deviation. Statistical significance was considered when *P*‐value was <.05. All experiments were conducted for at least three times.

## 3 RESULTS

### 3.1 LOXL1‐AS1 is upregulated in LUAD tissues and cells

**Figure 1:**
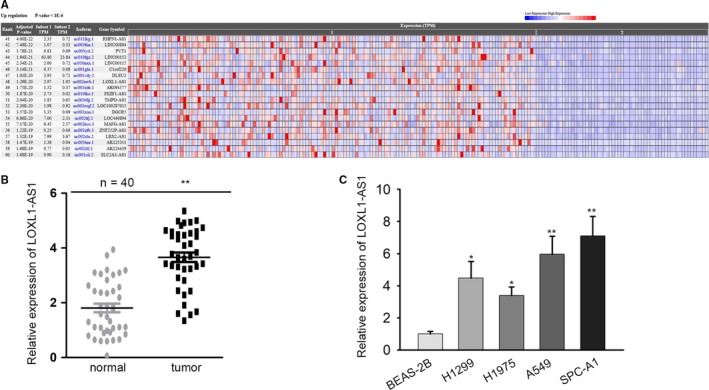
LOXL1‐AS1 is upregulated in LUAD tissues and cells. A, LOXL1‐AS1 expression in LUAD tissues was obtained from Cancer RNA‐Seq Nexus. B, LOXL1‐AS1 expression in paired LUAD and normal samples collected from 40 patients with LUAD. C, LOXL1‐AS1 expression in BEAS‐2B and human LUAD cells (H1299, H1975, A549, SPC‐A1) was detected by qRT‐PCR. Data were assessed under Student's *t* test. *P < .05, **P < .01 are statistical symbols that indicated the significant difference between groups. BEAS‐2B, human bronchial epithelial cell; LOXL1‐AS1, LOXL1 antisense RNA 1; LUAD, lung adenocarcinoma; qRT‐PCR, quantitative real‐time PCR

Through TCGA database, we found upregulated LOXL1‐AS1 in LUAD (Figure 1A). To examine whether LOXL1‐AS1 was associated with the tumorigenesis of LUAD, we first detected LOXL1‐AS1 expression in LUAD tissues. The matched adjacent normal tissues were used as a control. Results displayed that LOXL1‐AS1 was strongly overexpressed in LUAD tissues (Figure 1B). Consistently, data from qRT‐PCR manifested that LOXL1‐AS1 expression in LUAD cell lines (H1299, H1975, A549, SPC‐A1) was extremely higher than that in normal BEAS‐2B line (Figure 1C). Taken together, LOXL1‐AS1 was upregulated in LUAD tissues and cells.

### 3.2 LOXL1‐AS1 facilitates cell proliferation and migration as well as suppresses cell apoptosis

**Figure 2:**
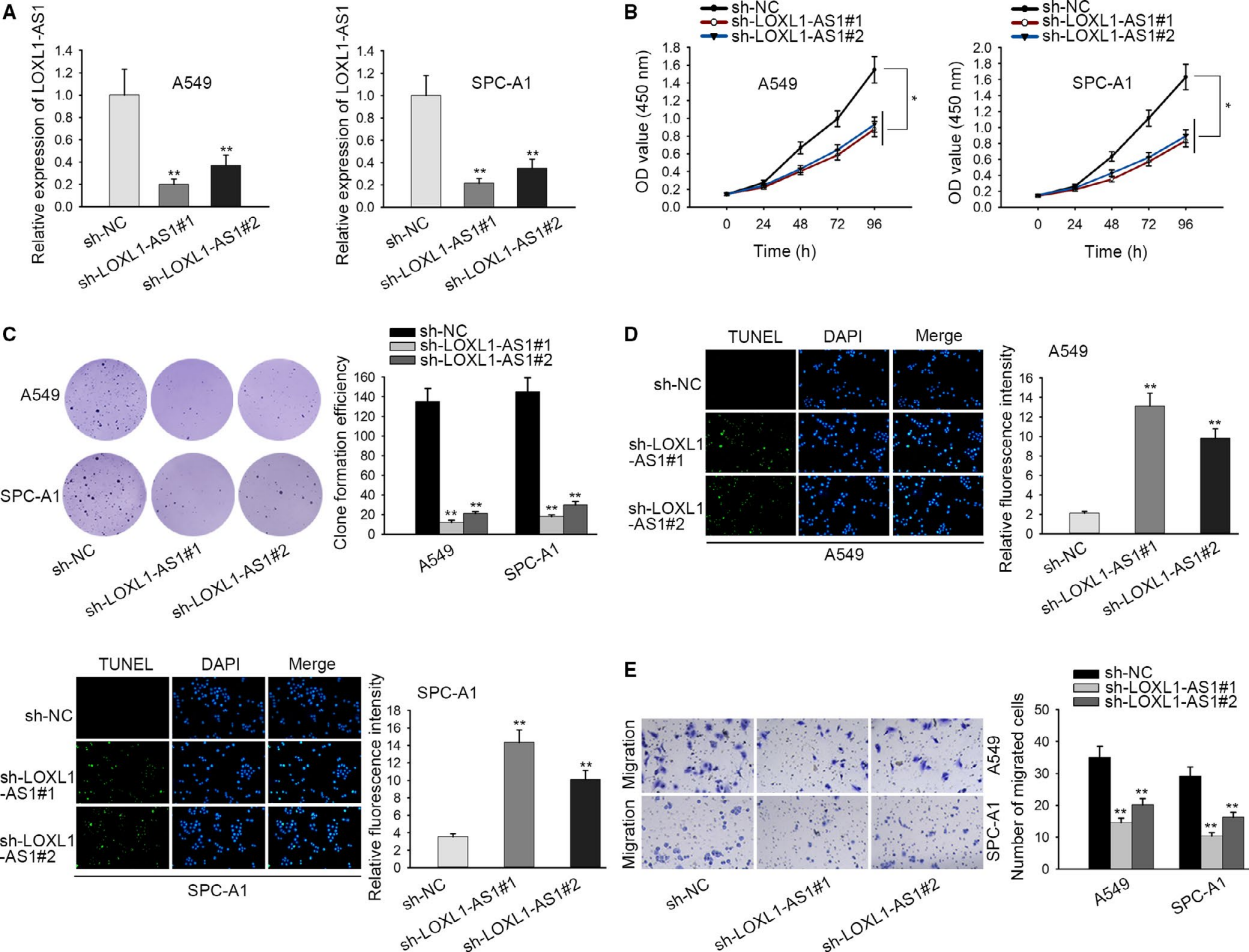
LOXL1‐AS1 facilitates cell proliferation and migration as well as suppresses cell apoptosis. A, qRT‐PCR was applied to examine the efficiency of LOXL1‐AS1 knockdown in A549 and SPC‐A1 cells. B and C, The proliferation in LOXL1‐AS1 silenced A549 and SPC‐A1 cells were accessed. D, The effect of LOXL1‐AS1 knockdown on the apoptosis of A549 and SPC‐A1 cells was estimated through TUNEL assay. E, Transwell assay was utilized to determine cell migration in LUAD cells transfected with sh‐LOXL1‐AS1. Data were assessed under Student's *t* test. *P < .05, **P < .01 are statistical symbols that indicated the significant difference between groups. LOXL1‐AS1, LOXL1 antisense RNA 1; LUAD, lung adenocarcinoma; qRT‐PCR, quantitative real‐time PCR

To explore the biological role of LOXL1‐AS1 in LUAD, shRNAs targeting LOXL1‐AS1 (sh‐LOXL1‐AS1#1, sh‐LOXL1‐AS1#2) were firstly transfected into A549 and SPC‐A1 cells. As a result, the expression of LOXL1‐AS1 was remarkably downregulated in sh‐LOXL1‐AS1 transfected A549 and SPC‐A1 cells (Figure 2A). Then, the effect of LOXL1‐AS1 knockdown on the proliferation of LUAD cells was tested by conducting CCK‐8 assay and colony formation assay. The results depicted that the proliferative ability of A549 and SPC‐A1 cells was significantly restrained by LOXL1‐AS1 silence (Figure 2B,C). Through TUNEL assay, it was identified that LOXL1‐AS1 deficiency efficaciously facilitated the apoptosis in A549 and SPC‐A1 cells (Figure 2D). Moreover, transwell assay was applied to measure cell migration, and the results revealed that the migratory capacity of LUAD cells was evidently inhibited with the transfection of sh‐LOXL1‐AS1 (Figure 2E). In short, LOXL1‐AS1 facilitates cell proliferation and migration as well as suppresses cell apoptosis in LUAD.

### 3.3 LOXL1‐AS1 acts as a sponge for miR‐423‐5p

**Figure 3:**
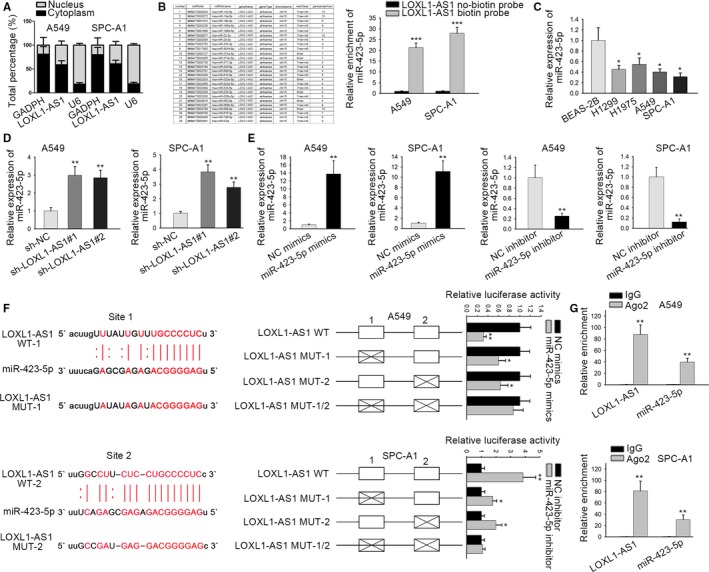
LOXL1‐AS1 acts as a sponge for miR‐423‐5p. A, Through nuclear‐cytoplasmic fractionation, the distribution of LOXL1‐AS1 in A549 and SPC‐A1 cells was evaluated. B, The potential target miRNAs of LOXL1‐AS1 were gained from starBase and screened out by RNA pull down assay. C, MiR‐423‐5p expression in BEAS‐2B and human LUAD cells (H1299, H1975, A549, SPC‐A1) was tested using qRT‐PCR. D, qRT‐PCR was employed to access miR‐423‐5p expression in cells transfected with sh‐LOXL1‐AS1. E, The transfection efficiency of miR‐423‐5p mimics (or inhibitor) was estimated by qRT‐PCR. F, The relationship between LOXL1‐AS1 and miR‐423‐5p was confirmed with the use of luciferase reporter assay. G, RIP assay was performed to verify direct interaction between LOXL1‐AS1 and miR‐423‐5p. Data were assessed under Student's *t* test. *P < .05, **P < .01, ***P < .001 are statistical symbols that indicated the significant difference between groups. BEAS‐2B, human bronchial epithelial cell; LOXL1‐AS1, LOXL1 antisense RNA 1; qRT‐PCR, quantitative real‐time PCR

To research the molecular mechanism of LOXL1‐AS1 in LUAD, we first determined the location of LOXL1‐AS1 in A549 and SPC‐A1 cells by using nuclear‐cytoplasmic fractionation. The results indicated that LOXL1‐AS1 was mainly distributed in the cytoplasm of LUAD cells (Figure 3A), indicating the regulatory role of LOXL1‐AS1 in posttranscription. Therefore, we speculated that LOXL1‐AS1 might function as a ceRNA to sponge certain miRNAs. Then, we searched potential miRNAs for LOXL1‐AS1, and 26 miRNAs that could bind to LOXL1‐AS1 were identified from starBase. Through RNA pull down assay, it was observed that miR‐423‐5p was strongly enriched in LOXL1‐AS1 biotin probe, while no significant changes were demonstrated in other miRNAs (Figure 3B). Besides, miR‐423‐5p expression was found to be prominently downregulated in LUAD cell lines with comparison to BEAS‐2B cell (Figure 3C). Besides, miR‐423‐5p expression was remarkably increased upon LOXL1‐AS1 knockdown (Figure 3D). In order to perform follow‐up assays, we elevated miR‐423‐5p expression with the transfection of miR‐423‐5p mimics. Additionally, we also weakened the expression of miR‐423‐5p by transfecting miR‐423‐5p inhibitor (Figure 3E). Furthermore, miR‐423‐5p was predicted to have two binding sites in LOXL1‐AS1, revealing that miR‐423‐5p was highly matched with the sequence of LOXL1‐AS1, which was subsequently verified by luciferase reporter assay (Figure 3F). RIP assay further validated that LOXL1‐AS1 and miR‐423‐5p were both significantly enriched in Ago2 antibodies, indicating the direct interaction between LOXL1‐AS1 and miR‐423‐5p (Figure 3F). These data delineated that LOXL1‐AS1 acted as a sponge of miR‐423‐5p.

### 3.4 MYBL2, a target gene of miR‐423‐5p, is modulated by LOXL1‐AS1

**Figure 4:**
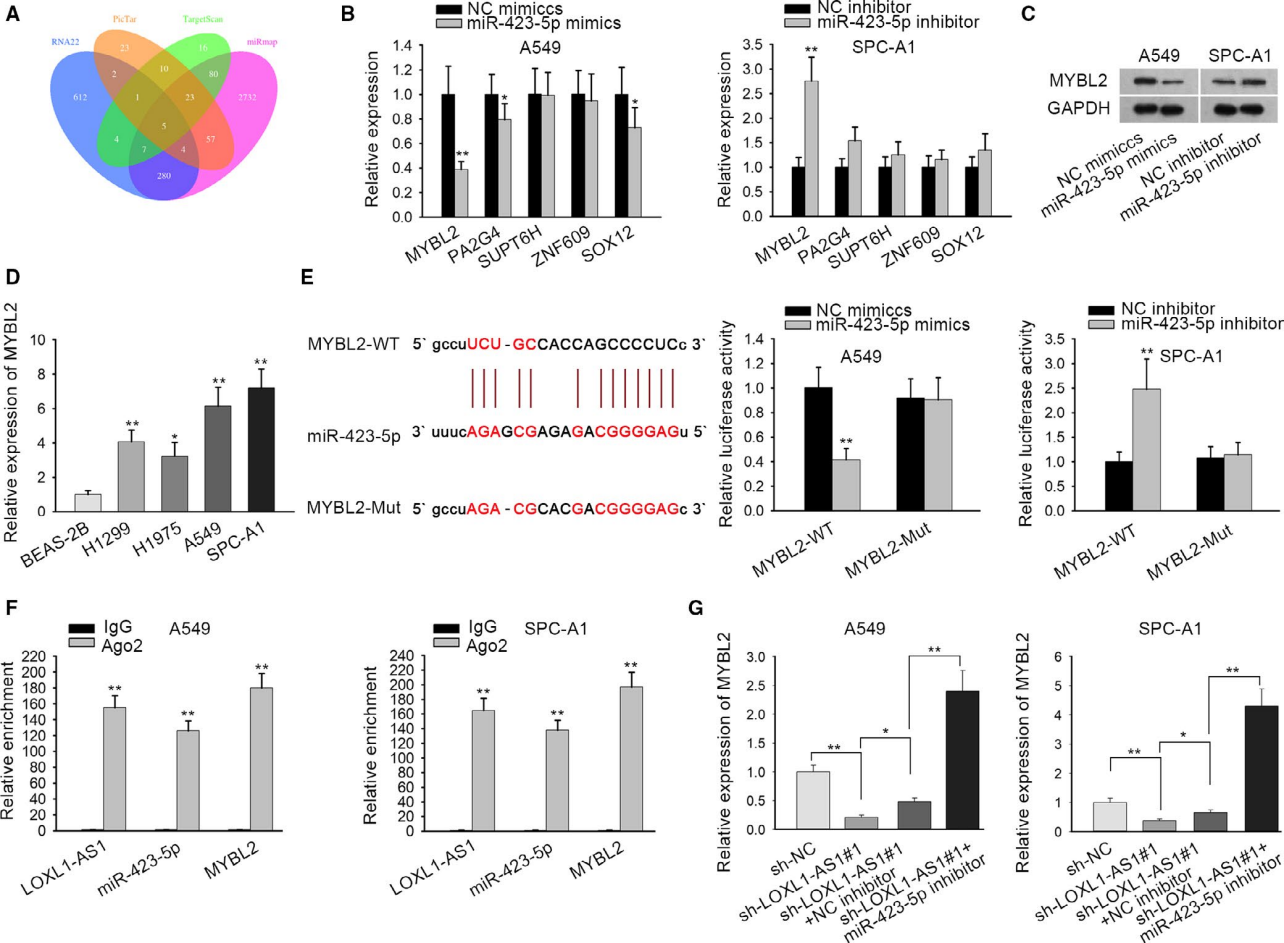
MYBL2, a target gene of miR‐423‐5p, is modulated by LOXL1‐AS1. A, The downstream target genes of miR‐423‐5p were shown as a form of Venn diagram according to PITA, RNA22, miRmap, and PicTar database. B, qRT‐PCR was conducted to examine the expressions of five potential mRNAs (MYBL2, PA2G4, SUPT6H, ZNF609 and SOX12) in cells transfected with miR‐423‐5p mimics (or inhibitor). C, mybl2 protein level in cells transfected with miR‐423‐5p mimics (or inhibitor) was detected by western blot assay. D, Relative expression of MYBL2 in BEAS‐2B and human LUAD cells (H1299, H1975, A549, SPC‐A1) was tested using qRT‐PCR. E, Luciferase reporter assay confirmed the interaction between miR‐423‐5p and MYBL2. F, miR‐423‐5p was interacted with LOXL1‐AS1 or MYBL2 was verified by performing RIP assay. G, qRT‐PCR analysis was utilized to estimate MYBL2 expression in sh‐NC, sh‐LOXL1‐AS1#1, sh‐LOXL1‐AS1#1+NC inhibitor and sh‐LOXL1‐AS1#1+miR‐423‐5p inhibitor groups. Data were assessed under Student's *t* test or one‐way ANOVA. *P < .05, **P < .01 are statistical symbols that indicated the significant difference between groups. BEAS‐2B, human bronchial epithelial cell; LOXL1‐AS1, LOXL1 antisense RNA 1; qRT‐PCR, quantitative real‐time PCR

To further support ceRNA hypothesis, we searched the downstream genes of miR‐423‐5p by bioinformatics analysis. Through RNA22, PicTar, TargetScan and miRmap, potential miR‐423‐5p target genes (MYBL2, PA2G4, SUPT6H, ZNF609, and SOX12) were presented by Venn diagram (Figure 4A). Results form qRT‐PCR analysis suggested that miR‐423‐5p potently affected the expression of MYBL2 among these candidate genes in A549 and SPC‐A1 cells (Figure 4B). According to western blot assay, we observed that MYBL2 protein level was decreased by miR‐423‐5p mimics and increased by miR‐423‐5p inhibitor (Figure 4C). Subsequently, MYBL2 expression was detected in LUAD cell lines. As illustrated in Figure 4D, MYBL2 mRNA expression was observably upregulated in LUAD cells in comparison with the control cell lines. Further, it was predicted that there was a binding site for miR‐423‐5p in MYBL2 3’ UTR, and luciferase reporter assay confirmed that the luciferase activity of WT MYBL2 was lessened with the transfection of miR‐423‐5p mimics, and strengthened by miR‐423‐5p inhibitor, whereas no significant difference was observed in that of mutant type MYBL2 (Figure 4E). In addition, RIP assay manifested that LOXL1‐AS1 and MYBL2 combined with the RISC which formed by miR‐423‐5p (Figure 4F). From Rescue assay, miR‐423‐5p inhibitor countervailed LOXL1‐AS1 knockdown‐mediated downregulation on mybl2 expression (Figure 4G). All the results manifested that MYBL2, the target gene of miR‐423‐5p, was modulated by LOXL1‐AS1.

### 3.5 LOXL1‐AS1/miR‐423‐5p/MYBL2 axis drives LUAD progression

**Figure 5:**
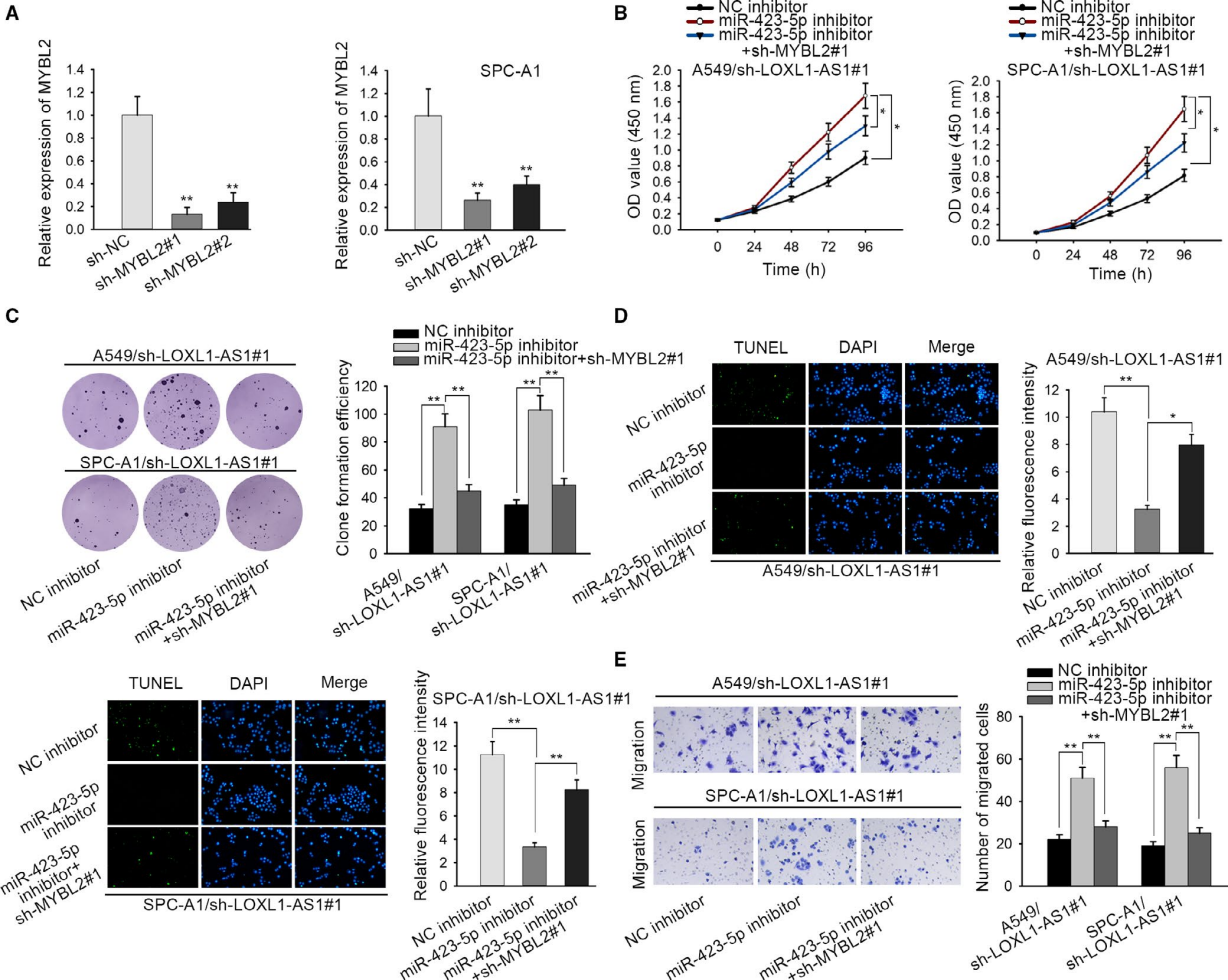
LOXL1‐AS1/miR‐423‐5p/MYBL2 axis drives LUAD progression. A, MYBL2 expression in sh‐MYBL2 transfected A549 and SPC‐A1 cells was tested by the use of qRT‐PCR. B and C, The proliferation of LOXL1‐AS1 silenced A549 and SPC‐A1 cells was evaluated after transfecting NC inhibitor, miR‐423‐5p inhibitor, miR‐423‐5p inhibitor + sh‐MYBL2#1 through CCK‐8 and colony formation assay. D, NC inhibitor, miR‐423‐5p inhibitor, miR‐423‐5p inhibitor+sh‐MYBL2#1 were transfected to LOXL1‐AS1 downregulated A549 and SPC‐A1 cells for observing cell apoptosis with TUNEL assay. E, Cell migration in A549 cell/sh‐LOXL1‐AS1#1 and SPC‐A1 cell/sh‐LOXL1‐AS1#1 transfected with NC inhibitor, miR‐423‐5p inhibitor, miR‐423‐5p inhibitor+sh‐MYBL2#1 was estimated with the employment of transwell assay. Data were assessed under one‐way ANOVA. *P < .05, **P < .01 are statistical symbols that indicated the significant difference between groups. CCK‐8, cell counting kit‐8; LOXL1‐AS1, LOXL1 antisense RNA 1; LUAD, lung adenocarcinoma; qRT‐PCR, quantitative real‐time PCR

To further probe that whether LOXL1‐AS1 enhanced LUAD development via miR‐423‐5p/MYBL2 axis, some restoration assays were designed and performed. Before the experiments, MYBL2 expression was knocked down in A549 and SPC‐A1 cells by transfecting sh‐MYBL2 (Figure 5A). Through CCK‐8 and colony formation assays, MYBL2 silence reversed the promotive effect of miR‐423‐5p inhibitor on the migration of LOXL1‐AS1 downregulated LUAD cells (Figure 5B,C). Besides, the apoptosis hampered by miR‐423‐5p inhibition was recovered with the transfection of sh‐MYBL2 in LOXL1‐AS1 silenced LUAD cells through TUNEL assay (Figure 5D). What's more, transwell assay depicted that miR‐423‐5p deficiency‐induced facilitation on cell migration was reserved by MYBL2 knockdown in LUAD cells transfected with sh‐LOXL1‐AS1 (Figure 5E). Namely, LOXL1‐AS1/miR‐423‐5p/MYBL2 axis could facilitate LUAD progression.

### 3.6 Transcription factor MYBL2 targets LOXL1‐AS1 promoter region

**Figure 6:**
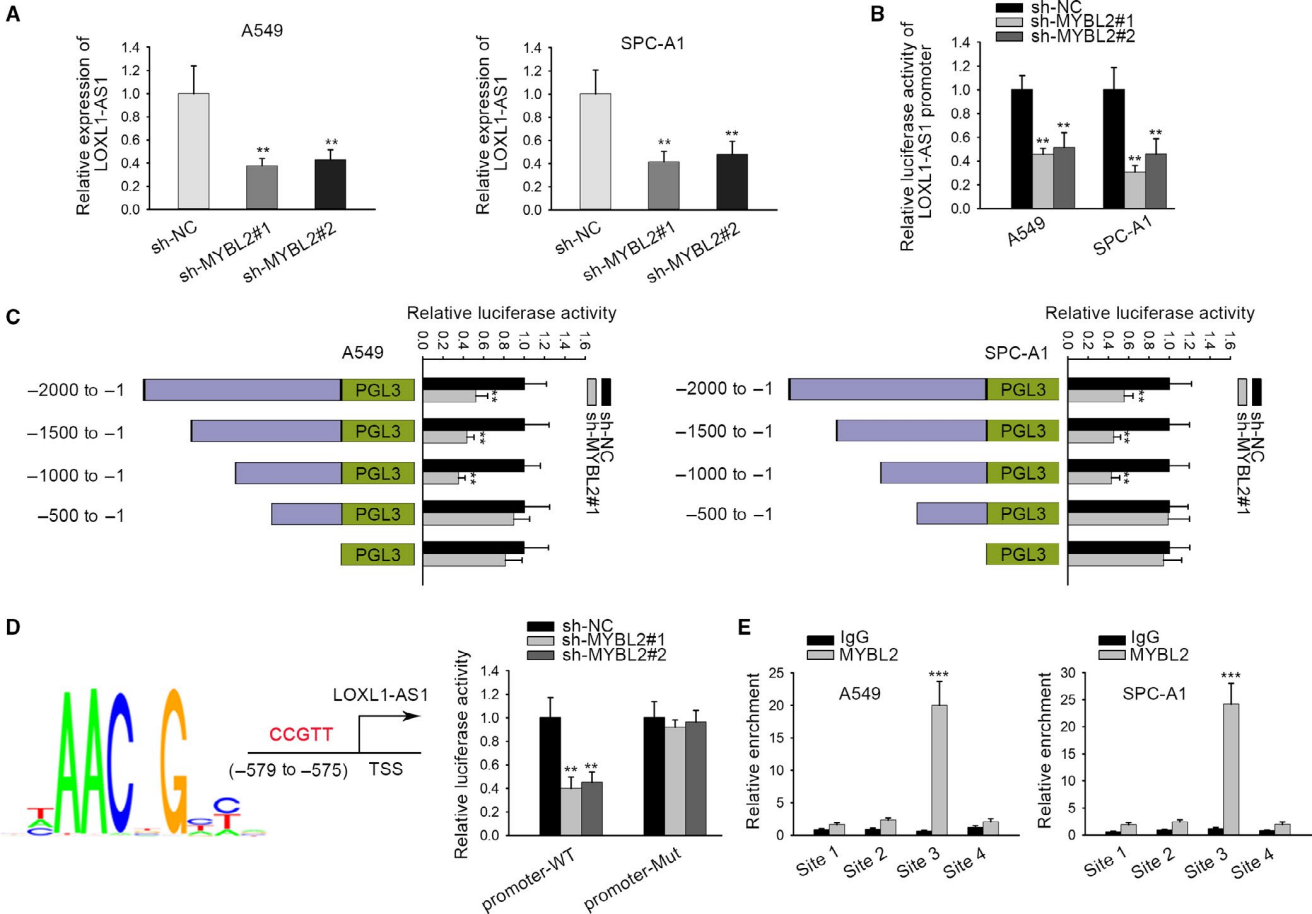
Transcription factor MYBL2 targets LOXL1‐AS1 promoter region. A, qRT‐PCR was conducted to detect the relative expression of LOXL1‐AS1 in sh‐MYBL2 transfected LUAD cells. B, Luciferase activity of LOXL1‐AS1 promoter upon MYBL2 knockdown was determined. C, The luciferase activity of P1, P2, P3, and P4 in LOXL1‐AS1 promoter region was evaluated by luciferase reporter assay. D, ChIP assay was applied to further confirm the interaction between MYBL2 and LOXL1‐AS1 promoter. E, MYBL2 was predicted to have a binding site in LOXL1‐AS1 promoter, which was subsequently validated by luciferase reporter assay. Data were compared under Student's *t* test. **P < .01, ***P < .001 are statistical symbols that indicated the significant difference between groups. ChIP, chromatin immunoprecipitation; LOXL1‐AS1, LOXL1 antisense RNA 1; LUAD, lung adenocarcinoma; qRT‐PCR, quantitative real‐time PCR

We had confirmed that LOXL1‐AS1/miR‐423‐5p/MYBL2 axis functioned as a critical regulation in LUAD. Interestingly, MYBL2 was reported to act as a transcription factor involved in the tumorigenesis of cancers.^16^, ^17^ Therefore, we speculated that MYBL2 could play a role of transcription factor to interact with LOXL1‐AS1 promoter. To confirm this, we detected the expression of LOXL1‐AS1 in sh‐MYBL2 transfected cells, and we found that MYBL2 silencing caused a repression on LOXL1‐AS1 expression (Figure 6A). Then, the results from luciferase reporter assay suggested that the knockdown of MYBL2 resulted in an evident decrease on the relative luciferase activity of LOXL1‐AS1 promoter (Figure 6B). To recognize the core promoter region of LOXL1‐AS1, four overlapped fragments of LOXL1‐AS1 promoter region were constructed, shown as P1 (−2000 to −1), P2 (−1500 to −1), P3 (−1000 to −1), and P4 (−500 to −1), and the luciferase activity in P1, P2, P3 was much higher than that in P4 (Figure 6C). Besides, four sites (site 1: −2000 to −1500, site 2: −1500 to −1000, site 3: −1000 to −500, site 4: −500 to −1) were marked in these fragments, and ChIP assay indicated that MYBL2 binds with LOXL1‐AS1 promoter region in site 3 (Figure 6D). As shown in Figure 6E, the binding site between MYBL2 and LOXL1‐AS1 promoter was predicted, and luciferase reporter assay revealed that the luciferase activity of WT LOXL1‐AS1 promoter was remarkably decreased upon MYBL2 knockdown. All data suggested that MYBL2 combined with LOXL1‐AS1 promoter.

## 4 DISCUSSION

In the past decades, accumulating numbers of studies have identified critical effects of lncRNAs on the carcinogenesis and progression of human tumors. LOXL1‐AS1, which researched in this study, has been reported to regulate prostate cancer cell cycle through sponging miR‐541‐3p and targeting CCND1.^18^ Moreover, it was also found to predict a poor prognosis and promote cell proliferation and metastasis in osteosarcoma.^19^ Nonetheless, its role in LUAD remains extremely unclear. In our study, a remarkable upregulation of LOXL1‐AS1 was discovered in LUAD tissues and cells. In addition, silenced LOXL1‐AS1 could effectively hamper cell proliferation and migration as well as enhance cell apoptosis in LUAD. All these results indicated the oncogenic role of LOXL1‐AS1 in LUAD.

MicroRNAs are small RNAs, which contain 21‐25 nucleotides,^20^ and correlated with the modulation of cellular processes, like cell proliferation,^21^ differentiation,^22^ and apoptosis.^23^ Through years of research, the dysregulation of miRNAs has been observed in multiple cancers, including LUAD.^24^ For example, miR‐767‐3p, an upstream gene of CLDN18, restrains cell growth and migration in LUAD.^25^ Through regulating AKT2, miR‐608 modulates the apoptosis of human LUAD cells.^26^ We also noticed that miR‐218 can inhibit EMT transition of LUAD cells via targeting Robo1 and Ecop.^27^ Increasing evidence suggested that lncRNA could act as a sponge of target miRNA to modulate cancer progression.^28^ For instance, lncRNA HNF1A‐AS1 sponges miR‐17‐5p to boost cell growth in non‐small cell lung cancer.^29^ LncRNA HOXD‐AS1, a sponge of miR‐133a‐3p, enhances cell proliferation and invasion via the activation of Wnt/β‐catenin signaling pathway in ovarian cancer.^30^ MiR‐423‐5p can be downregulated by lncRNA AFAP1‐AS1 and thereby facilitated the metastasis of nasopharyngeal carcinoma.^31^ However, its function and molecular mechanism in LUAD are largely obscure. Our present study indicated that miR‐423‐5p was considerably underexpressed in LUAD cells. Furthermore, its expression was negatively modulated by LOXL1‐AS1. Interestingly, two binding sites of miR‐423‐5p were confirmed in LOXL1‐AS1 sequence. Taken all together, LOXL1‐AS1 functioned as a sponge of miR‐423‐5p.

Emerging investigations have implied the carcinogenic function of MYBL2 in cancer progression,^32^ and it was also reported as a transcription factor in LUAD.^33^ Our study revealed that MYBL2 was targeted by miR‐423‐5p and LOXL1‐AS1 regulated the expression of MYBL2 by completely sponging miR‐423‐5p. Through rescue experiments, it was revealed that LOXL1‐AS1/miR‐423‐5p/MYBL2 axis could facilitate LUAD progression. In addition, MYBL2 was found to positively regulate the expression of LOXL1‐AS1 via inhibiting its promoter activity. This result indicated that MYBL2 could bind to LOXL1‐AS1 promoter, suggesting a positive feedback loop between LOXL1‐AS1 and MYBL2.

In conclusion, our data confirmed that lncRNA LOXL1‐AS1 functioned as an oncogene in LUAD, and regulated LUAD development thro ugh sponging miR‐423‐5p and targeting MYBL2. More importantly, MYBL2 could transcriptionally activate the expression of LOXL1‐AS1. These results disclosed LOXL1‐AS1/miR‐423‐5p/MYBL2 feedback loop in LUAD, providing a meaningful revelation for the exploration of LUAD diagnostic and therapeutic target.

## CONFLICT OF INTERESTS

Authors state no conflict of interest in this study.

## Data Availability

Research data and material are not shared.

## References

[1] Siegel RL , Miller KD , Jemal A . Cancer statistics, 2017. CA Cancer J Clin. 2017;67(1):7‐30. 28055103 10.3322/caac.21387

[2] Hasan N , Kumar R , Kavuru MS . Lung cancer screening beyond low‐dose computed tomography: the role of novel biomarkers. Lung. 2014;192(5):639‐648. 25108403 10.1007/s00408-014-9636-z

[3] Travis WD . The 2015 WHO classification of lung tumors. Pathologe. 2014;35(suppl 2):188. 25394966 10.1007/s00292-014-1974-3

[4] Gridelli C , Rossi A , Carbone DP , et al. Non‐small‐cell lung cancer. Nat Rev Dis Primers. 2015;1:15009. 27188576 10.1038/nrdp.2015.9

[5] Paez JG , Janne PA , Lee JC , et al. EGFR mutations in lung cancer: correlation with clinical response to gefitinib therapy. Science. 2004;304(5676):1497‐1500. 15118125 10.1126/science.1099314

[6] Colvin LA , Dougherty PM . Peripheral neuropathic pain: signs, symptoms, mechanisms, and causes: are they linked? Br J Anaesth. 2015;114(3):361‐363. 25253232 10.1093/bja/aeu323

[7] Ruan X . Long non‐coding RNA central of glucose homeostasis. J Cell Biochem. 2016;117(5):1061‐1065. 26530464 10.1002/jcb.25427

[8] Wang Y , Chen F , Zhao M , et al. The long noncoding RNA HULC promotes liver cancer by increasing the expression of the HMGA2 oncogene via sequestration of the microRNA‐186. J Biol Chem. 2017;292(37):15395‐15407. 28765279 10.1074/jbc.M117.783738 PMC5602398

[9] Liu C , Wang L , Li Y , Cui Y , Wang Y , Liu S . Long non‐coding RNA CHRF promotes proliferation and mesenchymal transition (EMT) in prostate cancer cell line PC3 requiring up‐regulating microRNA‐10b. Biol Chem. 2019;400:1035‐1045. 10.1515/hsz-2018-0380 30844757

[10] Meng L , Ma P , Cai R , Guan Q , Wang M , Jin B . Long noncoding RNA ZEB1‐AS1 promotes the tumorigenesis of glioma cancer cells by modulating the miR‐200c/141‐ZEB1 axis. Am J Transl Res. 2018;10(11):3395‐3412. 30662595 PMC6291700

[11] Ji N , Wang Y , Bao G , Yan J , Ji S . LncRNA SNHG14 promotes the progression of cervical cancer by regulating miR‐206/YWHAZ. Pathol Res Pract. 2019;215(4):668‐675. 30611620 10.1016/j.prp.2018.12.026

[12] Pan ZH , Guo XQ , Shan J , Luo SX . LINC00324 exerts tumor‐promoting functions in lung adenocarcinoma via targeting miR‐615‐5p/AKT1 axis. Eur Rev Med Pharmacol Sci. 2018;22(23):8333‐8342. 30556874 10.26355/eurrev_201812_16531

[13] Ye JJ , Cheng YL , Deng JJ , Tao WP , Wu L . LncRNA LINC00460 promotes tumor growth of human lung adenocarcinoma by targeting miR‐302c‐5p/FOXA1 axis. Gene. 2019;685:76‐84. 30359741 10.1016/j.gene.2018.10.058

[14] Liang R , Xiao G , Wang M , et al. SNHG6 functions as a competing endogenous RNA to regulate E2F7 expression by sponging miR‐26a‐5p in lung adenocarcinoma. Biomed Pharmacother. 2018;107:1434‐1446. 30257360 10.1016/j.biopha.2018.08.099

[15] Gao R , Zhang R , Zhang C , Liang Y , Tang W . LncRNA LOXL1‐AS1 promotes the proliferation and metastasis of medulloblastoma by activating the PI3K/AKT pathway. Anal Cell Pathol (Amst). 2018;2018:9275685. 30050750 10.1155/2018/9275685 PMC6040304

[16] Guan Z , Cheng W , Huang D , Wei A . High MYBL2 expression and transcription regulatory activity is associated with poor overall survival in patients with hepatocellular carcinoma. Curr Res Transl Med. 2018;66(1):27‐32. 29274707 10.1016/j.retram.2017.11.002

[17] Jia Y , Gao Y , Li J , Chang Z , Yan J , Qin Y . Prognostic implications of MYBL2 in resected Chinese gastric adenocarcinoma patients. Onco Targets Ther. 2019;12:1129‐1135. 30809094 10.2147/OTT.S188820 PMC6376880

[18] Long BO , Li NA , Xu X‐X , et al. Long noncoding RNA LOXL1‐AS1 regulates prostate cancer cell proliferation and cell cycle progression through miR‐541‐3p and CCND1. Biochem Biophys Res Commun. 2018;505(2):561‐568. 30278884 10.1016/j.bbrc.2018.09.160

[19] Chen S , Li W , Guo A . LOXL1‐AS1 predicts poor prognosis and promotes cell proliferation, migration, and invasion in osteosarcoma. Biosci Rep. 2019;39(4):BSR20190447. 30944201 10.1042/BSR20190447 PMC6488861

[20] Bartel DP . MicroRNAs: genomics, biogenesis, mechanism, and function. Cell. 2004;116(2):281‐297. 14744438 10.1016/s0092-8674(04)00045-5

[21] Liang B , Yin JJ , Zhan XR . MiR‐301a promotes cell proliferation by directly targeting TIMP2 in multiple myeloma. Int J Clin Exp Pathol. 2015;8(8):9168‐9174. 26464662 PMC4583894

[22] Antoniou A , Mastroyiannopoulos NP , Uney JB , Phylactou LA . miR‐186 inhibits muscle cell differentiation through myogenin regulation. J Biol Chem. 2014;289(7):3923‐3935. 24385428 10.1074/jbc.M113.507343 PMC3924261

[23] Guo J , Li M , Meng X , et al. MiR‐291b‐3p induces apoptosis in liver cell line NCTC1469 by reducing the level of RNA‐binding protein HuR. Cell Physiol Biochem. 2014;33(3):810‐822. 24685524 10.1159/000358654

[24] Zhang Z‐W , Chen J‐J , Xia S‐H , et al. Long intergenic non‐protein coding RNA 319 aggravates lung adenocarcinoma carcinogenesis by modulating miR‐450b‐5p/EZH2. Gene. 2018;650:60‐67. 29408583 10.1016/j.gene.2018.01.096

[25] Wan YL , Dai HJ , Liu W , Ma HT . miR‐767‐3p inhibits growth and migration of lung adenocarcinoma cells by regulating CLDN18. Oncol Res. 2018;26(4):637‐644. 29169410 10.3727/096504017X15112639918174 PMC7844711

[26] Othman N , Nagoor NH . miR‐608 regulates apoptosis in human lung adenocarcinoma via regulation of AKT2. Int J Oncol. 2017;51(6):1757‐1764. 29075783 10.3892/ijo.2017.4174

[27] Li YJ , Zhang W , Xia H , et al. miR‐218 suppresses epithelial‐to‐mesenchymal transition by targeting Robo1 and Ecop in lung adenocarcinoma cells. Future Oncol. 2017;13(28):2571‐2582. 28936884 10.2217/fon-2017-0398

[28] Yoon JH , Abdelmohsen K , Gorospe M . Functional interactions among microRNAs and long noncoding RNAs. Semin Cell Dev Biol. 2014;34:9‐14. 24965208 10.1016/j.semcdb.2014.05.015 PMC4163095

[29] Zhang G , An X , Zhao H , Zhang Q , Zhao H . Long non‐coding RNA HNF1A‐AS1 promotes cell proliferation and invasion via regulating miR‐17‐5p in non‐small cell lung cancer. Biomed Pharmacother. 2018;98:594‐599. 29289833 10.1016/j.biopha.2017.12.080

[30] Zhang Y , Dun Y , Zhou S , Huang XH . LncRNA HOXD‐AS1 promotes epithelial ovarian cancer cells proliferation and invasion by targeting miR‐133a‐3p and activating Wnt/beta‐catenin signaling pathway. Biomed Pharmacother. 2017;96:1216‐1221. 29239819 10.1016/j.biopha.2017.11.096

[31] Lian YU , Xiong F , Yang L , et al. Long noncoding RNA AFAP1‐AS1 acts as a competing endogenous RNA of miR‐423‐5p to facilitate nasopharyngeal carcinoma metastasis through regulating the Rho/Rac pathway. J Exp Clin Cancer Res. 2018;37(1):253. 30326930 10.1186/s13046-018-0918-9 PMC6191894

[32] Zhang K , Fu G , Pan G , et al. Demethylzeylasteral inhibits glioma growth by regulating the miR‐30e‐5p/MYBL2 axis. Cell Death Dis. 2018;9(10):1035. 30305611 10.1038/s41419-018-1086-8 PMC6180101

[33] Liu C , Zhang YH , Huang T , Cai Y . Identification of transcription factors that may reprogram lung adenocarcinoma. Artif Intell Med. 2017;83:52‐57. 28377053 10.1016/j.artmed.2017.03.010

